# Eicosa-11Z,14Z,17Z-Trienoic Acid Suppresses Melanogenesis by Inhibiting Intracellular MITF/Tyrosinase Axis

**DOI:** 10.4014/jmb.2604.04071

**Published:** 2026-10-08

**Authors:** Kyoung Mi Moon, Min-Kyeong Lee, Su-Yeon Park, Ah-reum Kim, Bonggi Lee

**Affiliations:** 1 Department of Food Science and Nutrition, Pukyong National University, Busan, Republic of Korea; 2 Biotechnology Research Division, National Institute of Fisheries Science, Busan, Republic of Korea; 3 Department of Smart Green Technology Engineering, Pukyong National University, Busan, Republic of Korea; 4 Marine Integrated Biomedical Technology Center, The National Key Research Institutes, Pukyong National University, Busan 48513, Republic of Korea

**Keywords:** Eicosa-11Z, 14Z, 17Z-trienoic acid, Melanogenesis, MITF, tyrosinase

## Abstract

Eicosa-11Z,14Z,17Z-trienoic acid (ETA; 20:3 n-3) is an ω-3 polyunsaturated fatty acid whose role in melanogenesis remains unclear. This study investigated the effects of ETA on melanogenesis using α-MSH-stimulated B16F10 melanoma cells and explored the underlying mechanisms. Structural similarity analysis indicated that ETA is closely related to α-linolenic acid, a lipid known to influence pigmentation. ETA showed minimal direct inhibition of mushroom tyrosinase activity in a cell-free system. In cell-based assays, ETA did not induce significant cytotoxicity within the tested concentration range. Under melanogenic conditions, ETA reduced α-MSH-induced melanin accumulation and intracellular tyrosinase activity. This reduction was accompanied by decreased mRNA expression of *Tyr*, *Tyrp1*, and *Tyrp2*, together with reduced protein expression of MITF, tyrosinase, TYRP1, and TYRP2. ETA also attenuated α-MSH-induced phosphorylation of CREB, ERK, and MITF. Immunofluorescence analysis further showed that ETA reduced α-MSH-induced MITF fluorescence intensity, including signals observed in the nuclear region. Taken together, these findings suggest that ETA modulates melanogenesis-related processes in B16F10 cells, likely through mechanisms involving changes in MITF localization and downstream gene expression rather than direct inhibition of tyrosinase activity. These results suggest that ETA may represent a potential candidate for further investigation in the context of pigmentation regulation.

## Introduction

Melanogenesis is a tightly regulated biosynthetic process that determines the color of skin, hair, and eyes and provides essential photoprotection against solar ultraviolet (UV) radiation [1]. Dysregulated or excessive melanin production contributes to a broad spectrum of pigmentary disorders, including melasma, solar lentigines, ephelides, and post-inflammatory hyperpigmentation, which can be cosmetically distressing and negatively affect quality of life [2]. In addition, chronic UV-driven melanogenesis is linked to photoaging and may participate in the pathogenesis of melanoma and other skin cancers, underscoring the need for safe and effective modulators of melanin synthesis [3].

Melanin is synthesized within specialized organelles termed melanosomes in epidermal melanocytes through a cascade of enzymatic reactions initiated by the rate-limiting oxidase tyrosinase [4]. Tyrosinase catalyzes the conversion of L-tyrosine to L-3,4-dihydroxyphenylalanine (L-DOPA) and subsequently to dopaquinone, which is further processed to form eumelanin and pheomelanin [1, 4]. Tyrosinase-related protein-1 (TYRP-1) and TYRP-2 (dopachrome tautomerase) participate in downstream steps that determine both the quantity and quality of melanin [5]. Expression of these melanogenic enzymes is orchestrated by the microphthalmia-associated transcription factor (MITF), a lineage-specific transcription factor that binds to E-box elements within the promoters of tyrosinase, TRP-1, and TRP-2 [6]. MITF integrates signals from multiple upstream pathways, including cyclic adenosine monophosphate (cAMP)/protein kinase A (PKA), mitogen-activated protein kinase/extracellular signal-regulated kinase (MAPK/ERK), and Wnt/β-catenin cascades, and its activity is critically determined by phosphorylation status and subcellular localization [7, 8]. Phosphorylated MITF translocates to the nucleus, where it promotes transcription of melanogenic genes and drives pigment production [8].

Most classical skin-lightening agents have been designed to directly inhibit tyrosinase catalytic activity [9]. Compounds such as kojic acid, arbutin, and hydroquinone can effectively reduce melanin synthesis; however, their clinical use is limited by issues of instability, cytotoxicity, irritancy, or safety concerns with long-term application [10, 11]. These limitations have prompted growing interest in strategies that target regulatory nodes upstream of tyrosinase, particularly MITF, thereby suppressing melanogenesis at the transcriptional level while potentially minimizing off-target toxicity [6, 7]. Recent evidence indicates that endogenous lipids, especially fatty acids of varying chain length and unsaturation, are important modulators of melanogenesis [12-14]. Saturated fatty acids like palmitic acid have been reported to enhance melanin production and stabilize tyrosinase, whereas mono- and polyunsaturated fatty acids such as palmitoleic acid, linoleic acid, and α-linolenic acid can down-regulate melanogenic signaling, promote tyrosinase degradation via the ubiquitin–proteasome system, and attenuate melanosome maturation [12, 15].

Work from our group and others has shown that certain structurally defined fatty acids suppress melanogenesis primarily by interfering with the intracellular MITF–tyrosinase axis rather than by directly blocking tyrosinase enzyme activity [13, 14]. For example, 10E-pentadecenoic acid (10E-PDA), a 15-carbon monounsaturated fatty acid, was recently reported to reduce melanin content and intracellular tyrosinase activity in α-melanocyte-stimulating hormone (α-MSH)–stimulated B16F10 melanoma cells without exhibiting strong inhibition of mushroom tyrosinase in a cell-free system [13]. Mechanistically, 10E-PDA decreased mRNA levels of *Tyr*, *Tyrp-1*, and *Tyrp-2*; lowered protein expression of Tyrosinase and TYRP-1; and impeded nuclear translocation of MITF, thereby attenuating MITF-dependent transcription [13]. Similarly, docosatrienoic acid (DTA; 22:3 n-3), a long-chain ω-3 polyunsaturated fatty acid, was shown to diminish melanin content and intracellular tyrosinase activity in B16F10 cells while only mildly inhibiting mushroom tyrosinase at high micromolar concentrations [14]. DTA down-regulated *Tyr*, *Tyrp-1*, and *Tyrp-2* mRNA levels and inhibited MITF nuclear accumulation, suggesting that modulation of MITF activity is the primary mechanism underlying its anti-melanogenic effects [16]. These findings collectively support the concept that specific unsaturated fatty acids can act as transcription-level regulators of melanogenesis.

Eicosa-11Z,14Z,17Z-trienoic acid (ETA; 20:3 n-3) is an ω-3 polyunsaturated fatty acid consisting of a 20-carbon chain with three cis double bonds located at the Δ11, Δ14, and Δ17 positions [17]. Although ETA shares key features with DTA and other bioactive ω-3 lipids, including a long, flexible acyl chain and multiple double bonds, it is distinct from previously studied anti-melanogenic fatty acids in carbon-chain length, ω-3 configuration, and double-bond position [14, 17]. ETA has been implicated in anti-inflammatory signaling and modulation of eicosanoid biosynthesis; however, its role in cutaneous biology, and in melanogenesis in particular, remains largely unexplored [17, 18]. Given the demonstrated anti-melanogenic properties of structurally related unsaturated fatty acids that target the MITF–Tyrosinase axis [13, 14], it is reasonable to hypothesize that ETA may exert similar regulatory effects on pigment production. However, because ETA differs from these previously examined fatty acids in terms of carbon-chain length, ω-3 configuration, and double-bond position, it should be evaluated as a distinct lipid candidate rather than as a direct extension of previously studied compounds.

In this study, we investigated whether ETA suppresses melanogenesis in α-MSH–stimulated B16F10 cells and examined whether this effect is associated with regulation of the MITF/tyrosinase axis. Melanin content, tyrosinase activity, MITF fluorescence intensity, and the mRNA and protein expression of MITF downstream targets, including tyrosinase, TYRP1, and TYRP2, were analyzed to determine whether ETA inhibits melanogenesis through intracellular regulatory mechanisms rather than direct tyrosinase inhibition.

## Materials and Methods

### Eicosa-11Z,14Z,17Z-trienoic acid (ETA)

Eicosa-11Z,14Z,17Z-trienoic acid (ETA; 20:3 n-3) was obtained from the SCREEN-WELL^®^ Fatty Acid Library (Enzo Life Sciences, USA; Cat. No. BML-2803-0100). ETA was supplied as a 10 mM stock solution in DMSO, stored at -20°C according to the manufacturer's instructions, and diluted to the indicated concentrations immediately before use.

### Structural Analysis Using SwissSimilarity

To investigate structural similarity, the SwissSimilarity web tool (http://www.swisssimilarity.ch/) [19] was utilized. The chemical structure of eicosatrienoic acid (ETA) was converted into SMILES format and used as the query molecule. Similarity screening was conducted against the ChEMBL (full database) [20] under the “Bioactive” compound class.

A 2D fingerprint-based method (FP2) was applied to evaluate structural similarity between ETA and compounds in the database. The retrieved compounds were ranked according to similarity scores, and top-ranking molecules were selected as structurally related compounds. The identities and structures of the selected compounds were further verified using the ChEMBL and PubChem databases prior to analysis.

### Cell-Free ROS Scavenging Assay

The ROS-scavenging activity of ETA was evaluated using a DCFH-DA fluorescence assay. Briefly, 10 μL of ETA (5–60 μM) or Trolox (15 μM, positive control) was added to each well of a black 96-well plate, followed by 10 μL of SIN-1 (10 μM) and 180 μL of 50 mM potassium phosphate buffer (PB, pH 7.4). DCFH-DA working solution was prepared by incubating DCFH-DA with esterase in the dark for 15–20 min and subsequently diluting the reaction mixture with PB. The working solution (50 μL) was then added to each well to achieve a final DCFH-DA concentration of 0.125 mM. Fluorescence was immediately monitored every 5 min for 30 min using Agilent BioTek Cytation 5 (Agilent Technologies, USA).

### Cell-free Peroxynitrite (ONOO^−^) Scavenging Assay

Peroxynitrite-scavenging activity was determined using a DHR123 fluorescence assay. Ten microliters of ETA (5–60 μM) or L-penicillamine (15 μM) was added to a black 96-well plate, followed by SIN-1 at a final concentration of 10 μM. After 1 h, 180 μL of a freshly prepared reaction mixture containing Rhodamine buffer, DTPA, and DHR123 was protected from light and added immediately before measurement. Fluorescence was immediately monitored every 5 min for 30 min using Agilent BioTek Cytation 5.

### Mushroom Tyrosinase Activity Assay

The effect of ETA on tyrosinase activity was evaluated using a cell-free mushroom tyrosinase assay adapted from previously reported methods [14]. ETA solutions (0, 5, 15, and 60 μM) were prepared in 50 mM sodium phosphate buffer (pH 6.5) containing 1 mM L-tyrosine. Kojic acid (15 μM) was used as a positive control for tyrosinase inhibition. The 0 μM ETA condition. The 0 μM condition contained DMSO alone as a vehicle control. The final concentration of DMSO did not exceed 1% (v/v) in all wells. Each reaction mixture was dispensed into a 96-well plate, and baseline absorbance at 490 nm was measured after brief shaking. Mushroom tyrosinase (1000 U/mL) was then added, and the reaction was carried out at 37°C under light-protected conditions. After incubation, absorbance was measured again at 490 nm, and enzyme activity was determined based on the change in absorbance.

### Cell Culture

B16F10 murine melanoma cells and HS68 human dermal fibroblasts were obtained from the American Type Culture Collection (ATCC, USA). HaCaT human keratinocytes were provided by the Korea Institute of Oriental Medicine (Republic of Korea). All cells were maintained in Dulbecco’s Modified Eagle’s Medium (DMEM) supplemented with heat-inactivated fetal bovine serum (FBS) and 1% penicillin–streptomycin. Cultures were incubated at 37°C in a humidified atmosphere containing 5% CO_2_.

### Cell Viability

Cell viability was assessed using an MTS-based assay (CellTiter96^®^ AQueous One Solution, Promega). B16F10, HS68, and HaCaT cells were seeded in 96-well plates at densities of 1 × 10^4^, 5 × 10^3^, and 5 × 10^3^ cells per well, respectively. After 24 h of incubation, cells were exposed to ETA at concentrations ranging from 1 to 100 μM in serum-free medium for an additional 24 h. The final DMSO concentration was adjusted to 1% (v/v) in all treatment groups, including the DMSO-only vehicle control. Following treatment, MTS reagent was added to each well and incubated for 1 h at 37°C in the dark. Absorbance was measured at 490 nm to determine relative cell viability.

### Intracellular ROS Assay

B16F10 cells were seeded in black 96-well plates at a density of 1 × 10^4^ cells/well in a total volume of 100 μL and cultured overnight. The cells were treated with ETA (5 and 15 μM) or Trolox (15 μM) for 24 h and subsequently exposed to SIN-1 (10 μM) to induce oxidative stress for 1 h. After treatment, the cells were washed once with phosphate-buffered saline (PBS), and 100 μL of PBS was added to each well. DCFH-DA working solution (125 μM) was freshly prepared in PBS immediately before use and protected from light. Subsequently, 50 μL of the DCFH-DA working solution was added to each well, and fluorescence was immediately monitored in kinetic mode every 5 min for 30 min using an Agilent BioTek Cytation 5 Cell Imaging Multi-Mode Reader.

### Intracellular Peroxynitrite (ONOO^-^) Assay

B16F10 cells were seeded in black 96-well plates at a density of 1 × 10^4^ cells/well in a total volume of 100 μL and cultured overnight. The cells were treated with ETA (5 and 15 μM) or L-penicillamine (15 μM) for 24 h, followed by treatment with SIN-1 (10 μM) for 1 h to induce oxidative stress. After treatment, the cells were washed once with phosphate-buffered saline (PBS), and 20 μL of PBS was added to each well. A DHR123 reaction mixture containing rhodamine buffer, DTPA, and DHR123 was freshly prepared immediately before use and protected from light. Subsequently, 180 μL of the reaction mixture was added to each well, and fluorescence was immediately monitored in kinetic mode every 5 min for 30 min using an Agilent BioTek Cytation 5 Cell Imaging Multi-Mode Reader.

### Measurement of Melanin Contents

Melanin levels were quantified using a modified version of a previously described method [14]. B16F10 cells were seeded in 6-well plates and allowed to attach for 24 h. Cells were pretreated with ETA (1, 5, or 15 μM) for 1 h before stimulation with α-MSH (500 nM). Vehicle (DMSO only) and α-MSH-treated cells served as controls. The final DMSO concentration was maintained at 0.15% (v/v) in all groups. Following 6 days of incubation, cells were harvested and lysed in 1 N NaOH, followed by heating at 60°C for 1 h to solubilize melanin. Absorbance was measured at 490 nm, and values were normalized based on protein content determined using a BCA assay.

### Intracellular Tyrosinase Activity Assay

Intracellular tyrosinase activity assays were measured as previously described with slight modifications [21]. B16F10 cells were pretreated with ETA (5 or 15 μM) or kojic acid (15 μM) for 1 h before stimulation with α-MSH (500 nM) and incubated for 6 days. The final DMSO concentration was adjusted to 0.15% (v/v) in all groups. Cells were lysed in sodium phosphate buffer (50 mM, pH 6.5) containing 1% Triton X-100 and 0.1 mM PMSF. After centrifugation (12,000 × g, 30 min, 4°C), the supernatant was collected and incubated with L-DOPA (2 mg/mL) at 37°C for 1 h. Enzymatic activity was determined by measuring absorbance at 490 nm.

### Real-Time PCR

Real-time PCR was performed using a previously described protocol [22]. Total RNA was extracted from B16F10 cells using RiboEX^TM^reagent (GeneAll). Complementary DNA (cDNA) was synthesized using the SmartGene Compact cDNA Synthesis Kit (SMART GENE). Quantitative PCR was performed using TOPreal^TM^ SYBR Green PreMIX (Enzynomics) on a QuantStudio^TM^ 1 Real-Time PCR system (Applied Biosystems). Relative gene expression levels were calculated using the 2^−ΔΔCt^ method, with normalization to an internal control gene.

### Western blot Analysis

B16F10 cells were seeded in 6 well plates. After 24 h of incubation, the cells were pretreated with ETA for 1 h and subsequently stimulated with α-MSH at the indicated concentrations for 4 days. After treatment, the cells were washed with cold phosphate-buffered saline (PBS) and lysed using ExKine^TM^ Total Protein Extraction Kit (KTP3006, Abbkine, USA) containing a protease inhibitor cocktail. Protein concentrations were determined using a BCA protein assay kit. Equal amounts of protein were separated by SDS-PAGE and transferred onto polyvinylidene difluoride (PVDF) membranes. The membranes were blocked with 5% skim milk in Tris-buffered saline containing 0.1% Tween-20 (TBST) and incubated overnight at 4°C with primary antibodies. After washing with TBST, the membranes were incubated with horseradish peroxidase-conjugated secondary antibodies for 1 h 30 min at room temperature. Protein bands were visualized using an enhanced chemiluminescence (ECL) detection reagent and captured with an imaging system (Azure 300, Azure Biosystems, USA). Band intensities were quantified using ImageJ software (National Institutes of Health, USA). Tyrosinase, TYRP1, TYRP2, and MITF protein levels were normalized to β-actin, whereas phosphorylated CREB, ERK, and MITF levels were normalized to their corresponding total protein levels.

### Immunofluorescence

Immunofluorescence staining was carried out to examine MITF localization [21]. B16F10 cells were fixed with 4% formalin and permeabilized using 0.1% Triton X-100. After blocking, cells were incubated overnight at 4°C with an anti-MITF primary antibody (1:100 dilution). Following washing, cells were incubated with a fluorescent secondary antibody (FSD^TM^ 488, 1:50 dilution) for 1 h at room temperature in the dark. Nuclei were counterstained with DAPI, and images were captured using a fluorescence microscope (LS40, LE.AM Solution Inc., Republic of Korea) under identical exposure settings for all groups to assess subcellular localization of MITF.

MITF fluorescence intensity was quantified using ImageJ software (version 1.53e; NIH). For nuclear MITF quantification, DAPI-positive nuclei were selected by thresholding the DAPI channel and converted into regions of interest. The nuclear regions of interest were then applied to the MITF channel, and the mean fluorescence intensity of MITF within the DAPI-defined nuclear area was measured after background subtraction. Background fluorescence was measured from cell-free regions in the same image and subtracted from each field. Ten randomly selected fields per group were analyzed from three independent experiments. Data were expressed as fold change relative to the untreated control.

### Statistics

Experiments were performed with the number of independent replicates indicated in each figure legend, and results are expressed as mean ± standard error of the mean (SEM). Unless otherwise stated, n indicates the number of independent biological replicates, not technical replicates. For cell experiments, biological replicates refer to independently prepared cell cultures for each treatment condition. The exact n is provided in each figure legend. Statistical analysis was conducted using GraphPad Prism (version 5.0; GraphPad Software, USA). Group differences were assessed by one-way analysis of variance (ANOVA) followed by Tukey’s multiple comparison test. A two-tailed p-value of less than 0.05 was considered statistically significant. Significance levels are indicated in the figures as *p < 0.05, **p < 0.01, and ***p < 0.001.

## Results

### Structural Features and Similarity Analysis of ETA

Eicosa-11Z, 14Z, 17Z-trienoic acid (ETA; 20:3 n-3) is an ω-3 polyunsaturated fatty acid characterized by a 20-carbon backbone with three cis double bonds at the Δ11, Δ14, and Δ17 positions [17]. This structural configuration, shared with other bioactive ω-3 lipids such as docosatrienoic acid (DTA), confers conformational flexibility that may influence membrane dynamics and lipid–protein interactions [14, 17]. To gain insight into the potential biological functions of ETA, we performed structural similarity analysis using the SwissSimilarity platform, which enables rapid comparison of compounds based on molecular fingerprints and three-dimensional similarity metrics [19]. This analysis revealed that ETA exhibits a high degree of structural similarity to α-linolenic acid, along with moderate similarity to other unsaturated fatty acids, including eicosapentaenoic acid, linoleic acid, dihomo-γ-linolenic acid, γ-linolenic acid, docosapentaenoic acid, and arachidonic acid (Fig. 1). Given that α-linolenic acid has been reported to suppress melanogenesis by reducing melanin production [12], these findings suggest that ETA may possess related functional properties. Based on this structural and functional association, we further investigated whether ETA exhibits modulatory effects on melanogenesis.

### Cell-Free Radical-Scavenging Activity of ETA and Its Effect on Mushroom Tyrosinase Activity

Oxidative and nitrosative stress, as well as antioxidant responses, have been reported to be associated with the regulation of melanogenesis [13, 14, 22]. Therefore, we first examined whether ETA exhibits ROS- and ONOO^−^-scavenging activity in cell-free assay systems. SIN-1 markedly increased ROS and ONOO?-associated fluorescence signals compared with the untreated control (Fig. 2A and 2B). Trolox, used as a positive antioxidant control, strongly reduced the SIN-1-induced ROS signal (Fig. 2A). ETA treatment also decreased the SIN-1-induced ROS signal at the tested concentrations, although the effect was weaker than that of Trolox (Fig. 2A). In the ONOO^−^ assay, L-penicillamine markedly suppressed the SIN-1-induced fluorescence signal, confirming the responsiveness of the assay system (Fig. 2B). ETA produced a modest reduction in the ONOO^−^ -associated signal, with a significant decrease observed at the highest concentration tested (Fig. 2B). These results suggest that ETA exhibits limited but detectable radical-scavenging activity under cell-free conditions.

We next examined whether ETA directly inhibits tyrosinase catalytic activity using a mushroom tyrosinase assay. As expected, kojic acid significantly inhibited mushroom tyrosinase activity, confirming the validity of the assay. In contrast, ETA showed little to no inhibitory effect on mushroom tyrosinase activity across the tested concentration range (Fig. 2C). These findings suggest that the anti-melanogenic effect of ETA is unlikely to be primarily attributable to direct inhibition of tyrosinase enzymatic activity.

### ETA Reduces Melanin Accumulation and Intracellular Tyrosinase Activity

To further define the in vitro activity profile of ETA, we first examined its effects on cell viability in three skin-related cell lines, namely B16F10 melanoma cells, HS68 dermal fibroblasts, and HaCaT keratinocytes. Across the concentration range tested, ETA did not produce significant cytotoxicity within the doses used for the subsequent assays (Fig. 3A-3C). Viability was preserved up to 15 μM in B16F10 cells, up to 100 μM in HS68 cells, and up to 50 μM in HaCaT cells (Fig. 3A-3C). We next examined whether ETA influences pigment production under melanogenic conditions. B16F10 cells were pre-treated with ETA prior to stimulation with α-MSH to induce melanogenesis. As expected, α-MSH markedly increased melanin accumulation compared with the untreated control, confirming effective induction of the melanogenic response (Fig. 3F). Under these conditions, ETA reduced the α-MSH-induced increase in melanin content (Fig. 3F). At 1 μM, ETA decreased melanin levels by 37.6%, and a greater reduction of up to 48.0% was observed at 5 μM. Increasing the concentration to 15 μM did not result in a further decrease (Fig. 3F). A comparable pattern was observed in the intracellular tyrosinase activity assay. Tyrosinase activity was elevated following α-MSH stimulation, whereas ETA pre-treatment diminished this increase (Fig. 3G). The concurrent decrease in melanin content and intracellular tyrosinase activity indicates that ETA treatment was associated with reduced melanogenic activity within the cells.

### ETA Alters the Expression of Melanogenesis-Related Genes in α-MSH-Stimulated B16F10 Cells

To further assess whether these changes were associated with transcriptional regulation, we analyzed the mRNA expression of representative melanogenesis-related genes, including *Tyrp1*, *Tyrp2*, *Tyr*, *Creb1*, and *Mc1r* (Fig. 4). The expression patterns of these genes were not affected to the same extent. ETA treatment produced little change in *Creb1* and *Mc1r* expression (Fig. 4D-4E), indicating that their mRNA levels were not markedly altered under the conditions tested. In contrast, the α-MSH-induced elevation of *Tyrp1*, *Tyrp2*, and *Tyr* mRNA levels was clearly reduced in the presence of ETA (Fig. 4A-4C). Among these melanogenic markers, tyrosinase showed the strongest response to ETA (Fig. 4C), exhibiting the largest decrease relative to the α-MSH-treated group. Taken together, these results show that ETA reduced melanin accumulation and intracellular tyrosinase activity in α-MSH-stimulated B16F10 cells, accompanied by lowered expression of key melanogenesis-related genes, particularly *Tyr*.

### ETA Suppresses Melanogenesis-Related Protein Expression and Phosphorylation Signaling in α-MSH-Stimulated B16F10 Cells

To further validate whether the transcriptional changes were reflected at the protein level, we examined the expression of melanogenesis-related proteins by Western blot analysis. α-MSH stimulation increased the protein levels of tyrosinase, TYRP1, TYRP2, and MITF compared with the untreated control (Fig. 5A and 5C). ETA treatment reduced the α-MSH-induced increases in these melanogenic proteins, with a more pronounced decrease observed at 15 μM ETA (Fig. 5A and 5C). These results support the qPCR findings and suggest that ETA-mediated suppression of melanogenesis is associated with reduced expression of MITF and its downstream melanogenic targets at the protein level.

Because MITF activity is regulated not only by expression level but also by phosphorylation-dependent signaling pathways, we further examined the phosphorylation status of CREB, ERK, and MITF. α-MSH increased the phosphorylation levels of CREB, ERK, and MITF compared with the untreated control (Fig. 5B and 5D). ETA treatment attenuated these phosphorylation signals, particularly at 15 μM, as shown by reduced p-CREB/CREB, p-ERK/ERK, and p-MITF/MITF ratios (Fig. 5B and 5D). These findings are consistent with the possibility that ETA affects α-MSH-induced melanogenic signaling at both the protein-expression level and the phosphorylation-dependent signaling level.[Table T1]

### ETA Treatment Attenuates α-MSH-Induced MITF Accumulation in B16F10 Cells

Because MITF functions as a central regulator of melanogenic gene expression, we next examined MITF fluorescence signals using immunofluorescence analysis. Based on the melanin and tyrosinase activity results, 5 μM ETA was selected for subsequent imaging experiments, as this concentration showed a marked anti-melanogenic effect under the tested conditions. In B16F10 cells, α-MSH stimulation increased MITF fluorescence intensity compared with the untreated control. In contrast, cells pre-treated with ETA displayed a noticeable reduction in MITF fluorescence intensity, including signals observed in the nuclear region (Fig. 6A). Quantitative analysis further confirmed that ETA significantly reduced the α-MSH-induced increase in MITF fluorescence intensity (Fig. 6B). These findings suggest that ETA suppresses α-MSH-induced MITF accumulation, which is consistent with the reduced expression of melanogenesis-related genes and proteins observed under the same conditions.

## Discussion

The present study shows that ETA exerts a significant anti-melanogenic effect in α-MSH–stimulated B16F10 melanoma cells while exhibiting minimal cytotoxicity within the concentration range tested. ETA markedly reduced total melanin content and intracellular tyrosinase activity, yet showed little to no inhibitory activity against mushroom tyrosinase in a cell-free system, even at comparatively high concentrations. This dissociation between strong cellular effects and weak direct enzyme inhibition parallels our previous observations with 10E-PDA and DTA [13, 14], and suggests that ETA-mediated suppression of melanogenesis is more closely associated with intracellular regulatory events than with direct inhibition of tyrosinase enzymatic activity.

Because oxidative and nitrosative stress have been associated with melanogenesis regulation [13, 14, 22], we evaluated the effects of ETA on ROS- and ONOO^−^-associated fluorescence signals. ETA reduced ROS- and ONOO^−^-associated signals in both cell-free assays and B16F10 cells, indicating that ETA can modulate oxidative and nitrosative stress-related responses under the present experimental conditions. In addition, because ETA is a polyunsaturated fatty acid that may be susceptible to autoxidation, we examined whether antioxidant co-treatment altered its anti-melanogenic effect. Co-treatment with Trolox did not reverse ETA-mediated suppression of α-MSH-induced melanin accumulation (Supplementary Fig. S1), suggesting that the observed effect is unlikely to be solely attributable to oxidation-derived products. However, these redox-related findings do not by themselves establish antioxidant regulation as the primary mechanism of ETA-mediated melanogenesis suppression. Direct chemical analyses of ETA oxidation status, such as peroxide value or conjugated-diene measurements, were not performed in the present study and should be addressed in future work.

At the molecular level, ETA treatment down-regulated the mRNA expression of melanogenesis-related genes, including *Tyr*, *Tyrp1*, and *Tyrp2*, and concomitantly reduced MITF expression and MITF-associated phosphorylation signaling, suggesting that ETA-mediated melanogenesis suppression is associated with regulation of the MITF-centered melanogenic network. Western blot analysis further showed that ETA reduced α-MSH-induced protein expression of MITF, tyrosinase, TYRP1, and TYRP2, supporting the qPCR and immunofluorescence results. MITF is widely recognized as a master regulator of melanocyte lineage specification, survival, and pigment production, controlling transcription of *Tyr*, *Tyrp1*, *Tyrp2*, and other melanogenic genes [7, 8]. Consistent with findings for DTA and 10E-PDA [13, 14], in which inhibition of MITF activation led to decreased expression of melanogenic enzymes, our data suggest that ETA treatment is associated with reduced MITF-dependent melanogenic signaling and decreased pigment synthesis. Because MITF activity is regulated by multiple upstream pathways, including cAMP/PKA, MAPK/ERK, and PI3K/AKT signaling [7, 8], we further examined phosphorylation-dependent signaling events. ETA attenuated α-MSH-induced phosphorylation of CREB, ERK, and MITF. These findings indicate that ETA affects multiple components of melanogenic signaling; however, they do not establish that the alteration in ERK phosphorylation directly mediates the observed changes in MITF phosphorylation or activity. Further studies using pathway-specific inhibitors and time-course analyses are needed to clarify the relationship between ERK and MITF in the response to ETA. Therefore, the present data support an association between ETA treatment and suppression of MITF/tyrosinase-axis signaling, rather than fully defining the upstream molecular mechanism.

The observation that ETA lowers intracellular tyrosinase activity without directly inhibiting the enzyme in vitro raises the possibility that decreased enzyme abundance and/or altered post-translational handling rather than catalytic blockade is responsible for the functional outcome. Unsaturated fatty acids, including linoleic and α-linolenic acids, have been reported to enhance ubiquitin–proteasome–mediated degradation of tyrosinase, disrupt melanosomal maturation, and modify membrane microdomain composition, thereby reducing the amount of active tyrosinase delivered to melanosomes [12, 15]. Our qPCR data showing diminished tyrosinase mRNA in ETA-treated cells indicate that transcriptional down-regulation is at least one contributor to reduced enzyme activity. However, whether ETA also affects tyrosinase protein stability, ubiquitination, or intracellular trafficking was not directly examined in the present study. Future studies employing pulse–chase analyses, assessment of tyrosinase ubiquitination, and subcellular fractionation of melanosomes will be necessary to determine whether ETA engages additional post-transcriptional mechanisms akin to other anti-melanogenic fatty acids.

From a structural standpoint, ETA belongs to the family of long-chain ω-3 polyunsaturated fatty acids characterized by multiple cis double bonds that confer high conformational flexibility and a propensity to incorporate into phospholipid bilayers [17, 18]. Although ETA showed partially overlapping biological outcomes with previously reported anti-melanogenic fatty acids, it is structurally distinct from 10E-PDA and DTA. 10E-PDA is a C15 monounsaturated fatty acid, DTA is a C22 trienoic fatty acid, and ETA is a C20 ω-3 polyunsaturated fatty acid with three cis double bonds at the Δ11, Δ14, and Δ17 positions. Thus, the present study extends our previous observations by showing that a structurally distinct C20 ω-3 PUFA can attenuate α-MSH-induced melanogenesis.

DTA, which was recently shown to inhibit melanogenesis by suppressing MITF nuclear translocation and melanogenesis-related gene expression, has a related trienoic fatty acid framework but differs from ETA in carbon-chain length [14]. Bioinformatic analyses using SwissSimilarity previously revealed that DTA clusters with several known anti-melanogenic lipids, including palmitoleic acid, linoleic acid, and α-linolenic acid, suggesting a possible structure–activity relationship in which specific patterns of unsaturation and chain length may contribute to anti-melanogenic activity [14]. In this context, the present findings with ETA further support the possibility that structurally related unsaturated fatty acids can converge on melanogenic regulation despite differences in carbon-chain length and double-bond position. However, SwissSimilarity-based molecular fingerprint analysis should be interpreted as a preliminary structural comparison, because it does not fully account for fatty-acid stereochemistry, conformational flexibility, membrane behavior, or cellular metabolism. Therefore, the precise molecular target of ETA remains to be identified, and future studies comparing ETA, DTA, 10E-PDA, and other ω-3/ω-6 analogues will be necessary to clarify how fatty-acid structure relates to melanogenic regulation.

Another noteworthy aspect is the potential multifunctionality of ETA in skin. Fatty acids and their derivatives are key components of the epidermal barrier, contributing to stratum corneum lipid organization, transepidermal water loss control, and resilience against environmental stressors [23]. In cosmetic and dermatological formulations, fatty acids can act not only as biologically active ingredients but also as emollients, penetration enhancers, and stabilizers that improve product texture and delivery of co-actives [23, 24]. Although ETA reduced ROS and ONOO?-associated signals in cell-free assays and B16F10 cells, these redox-related findings do not by themselves establish antioxidant regulation as the primary mechanism of ETA-mediated melanogenesis suppression. Therefore, the broader dermatological and cosmetic relevance of ETA should be considered preliminary. Future studies using keratinocytes, dermal fibroblasts, melanocyte–keratinocyte co-culture systems, and three-dimensional skin models will be necessary to further evaluate the broader effects of ETA on skin homeostasis and barrier function.

Several limitations of this study should be acknowledged. First, our experiments were confined to the murine B16F10 melanoma cell line, which, although widely used as a melanogenesis model, does not fully recapitulate the complexity of human epidermal melanocyte–keratinocyte interactions. Validation of ETA’s anti-melanogenic efficacy and safety in primary human melanocytes, co-culture systems, and reconstructed human skin models will be required to translate these findings toward clinical or cosmetic applications. Second, although ETA reduced MITF and melanogenic enzyme expression at both the mRNA and protein levels and attenuated phosphorylation of CREB, ERK, and MITF, we did not fully delineate the specific upstream signaling pathway responsible for these effects. In light of the documented roles of MAPK/ERK, PI3K/AKT, and cAMP/PKA pathways in regulating MITF activity [7, 8], future work employing selective kinase inhibitors, phospho-specific antibodies, and time-course analyses will be valuable to map ETA’s position within the melanogenic signaling network. Third, direct chemical analyses of ETA oxidation status, such as peroxide value or conjugated-diene measurements, were not performed in the present study and should be addressed in future work. Fourth, pharmacokinetic properties such as skin penetration, metabolic stability, and potential systemic exposure were not addressed and should be examined in appropriate in vivo models.

Despite these limitations, our data suggest that eicosa-11Z,14Z,17Z-trienoic acid is a novel ω-3 fatty acid that attenuates melanogenesis, at least in part, through regulation of the MITF–tyrosinase axis rather than by directly inhibiting tyrosinase enzyme activity. Together with prior evidence for 10E-PDA and DTA [13, 14], the present findings support the possibility that structurally defined unsaturated fatty acids can function as transcriptional modulators of pigment production. ETA’s combination of transcription-level control of melanogenic genes, lack of pronounced direct tyrosinase inhibition, and potential lipid-based properties may make it a candidate for further investigation as a pigmentation-modulating ingredient. Further mechanistic and translational studies are warranted to further clarify the therapeutic and cosmetic potential of this lipid mediator.

## Supplemental Materials

Supplementary data for this paper are available on-line only at http://jmb.or.kr.



## Figures and Tables

**Fig. 1:**
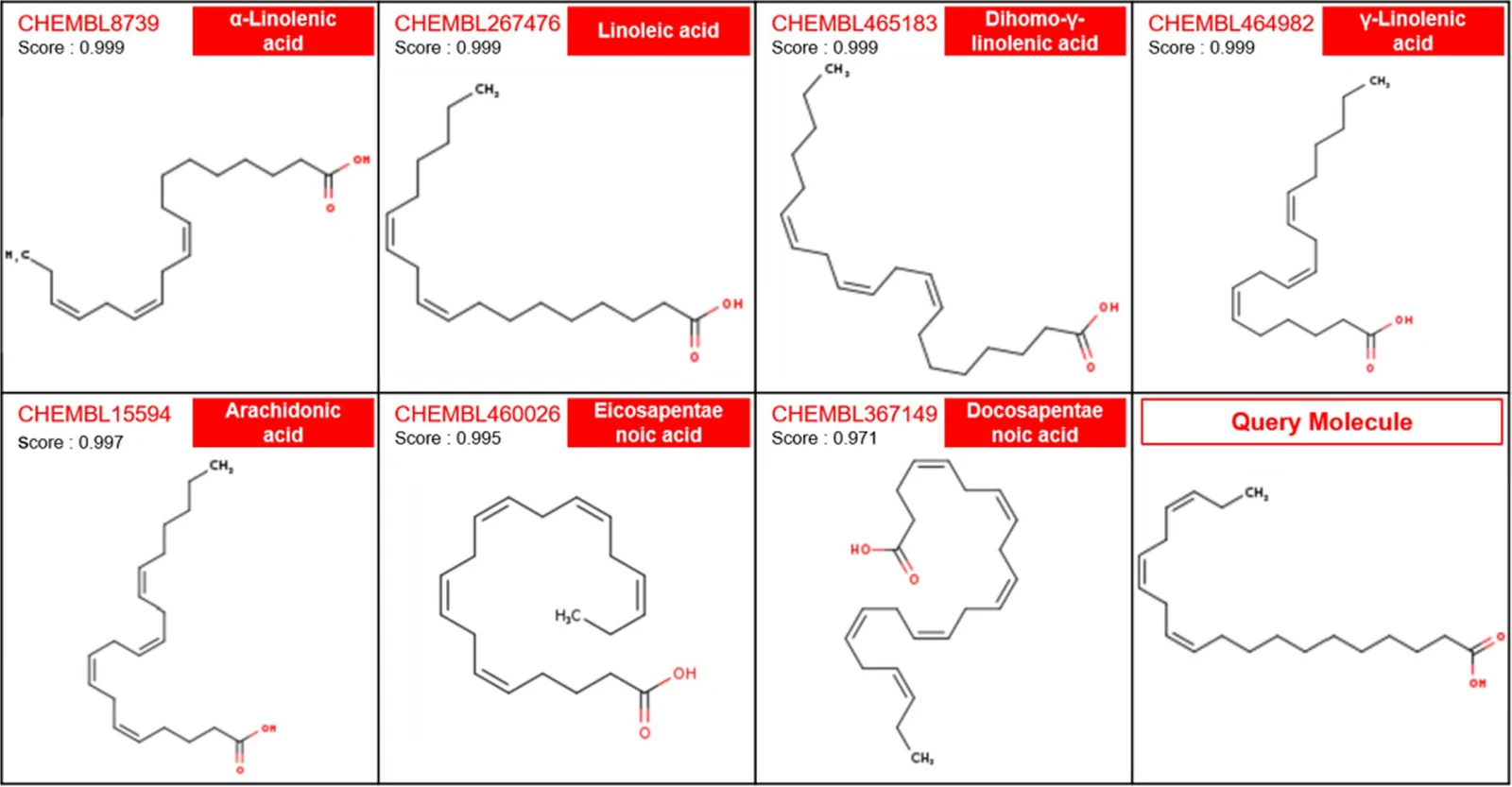
Structural similarity analysis of ETA with related compounds. SwissSimilarity was used to identify compounds structurally similar to ETA using molecular fingerprint and 3D shape similarity methods. Structurally related compounds were retrieved from the ChEMBL database and ranked based on similarity scores. Representative long-chain fatty acids are presented, including α-linolenic acid, eicosapentaenoic acid, linoleic acid, dihomo-γ-linolenic acid, γ-linolenic acid, docosapentaenoic acid, and arachidonic acid.

**Fig. 2:**
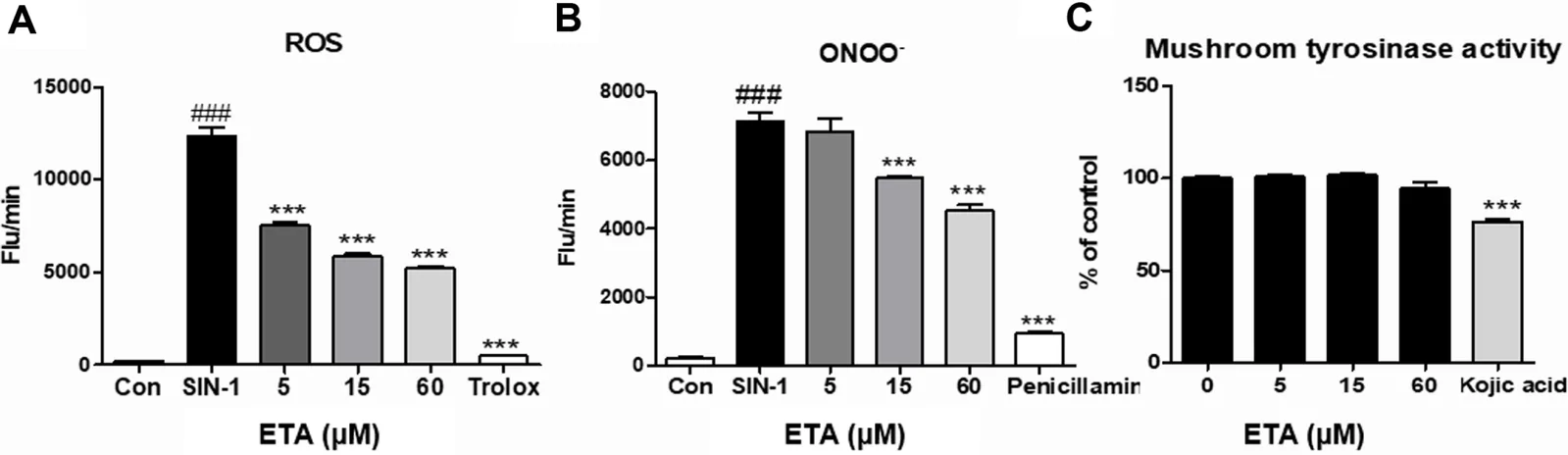
ROS- and ONOO^−^-scavenging activities and tyrosinase inhibitory activity of ETA in cell-free assays. (**A**) Reactive oxygen species (ROS)-scavenging activity of ETA (5−60 μM) or Trolox (15 μM) was evaluated using a DCFH-DA-based assay with SIN-1 (10 μM) (n = 8). (**B**) Peroxynitrite (ONOO^−^)-scavenging activity of ETA (5−60 μM) or L-penicillamine (15 μM) was determined using a DHR123 fluorescence assay with SIN-1 (10 μM) (n = 10). Statistical significance was indicated as *** *p* < 0.001 vs. SIN-1-treated group; ### *p* < 0.001 vs. untreated control. (**C**) Tyrosinase inhibitory activity of ETA was assessed using mushroom tyrosinase, with kojic acid (15 μM) as a positive control (n = 3). Statistical significance was indicated as *** *p* < 0.001 vs. untreated control. Data are presented as mean ± SEM. All statistical significance was determined by one-way ANOVA followed by Tukey's multiple comparisons test.

**Fig. 3:**
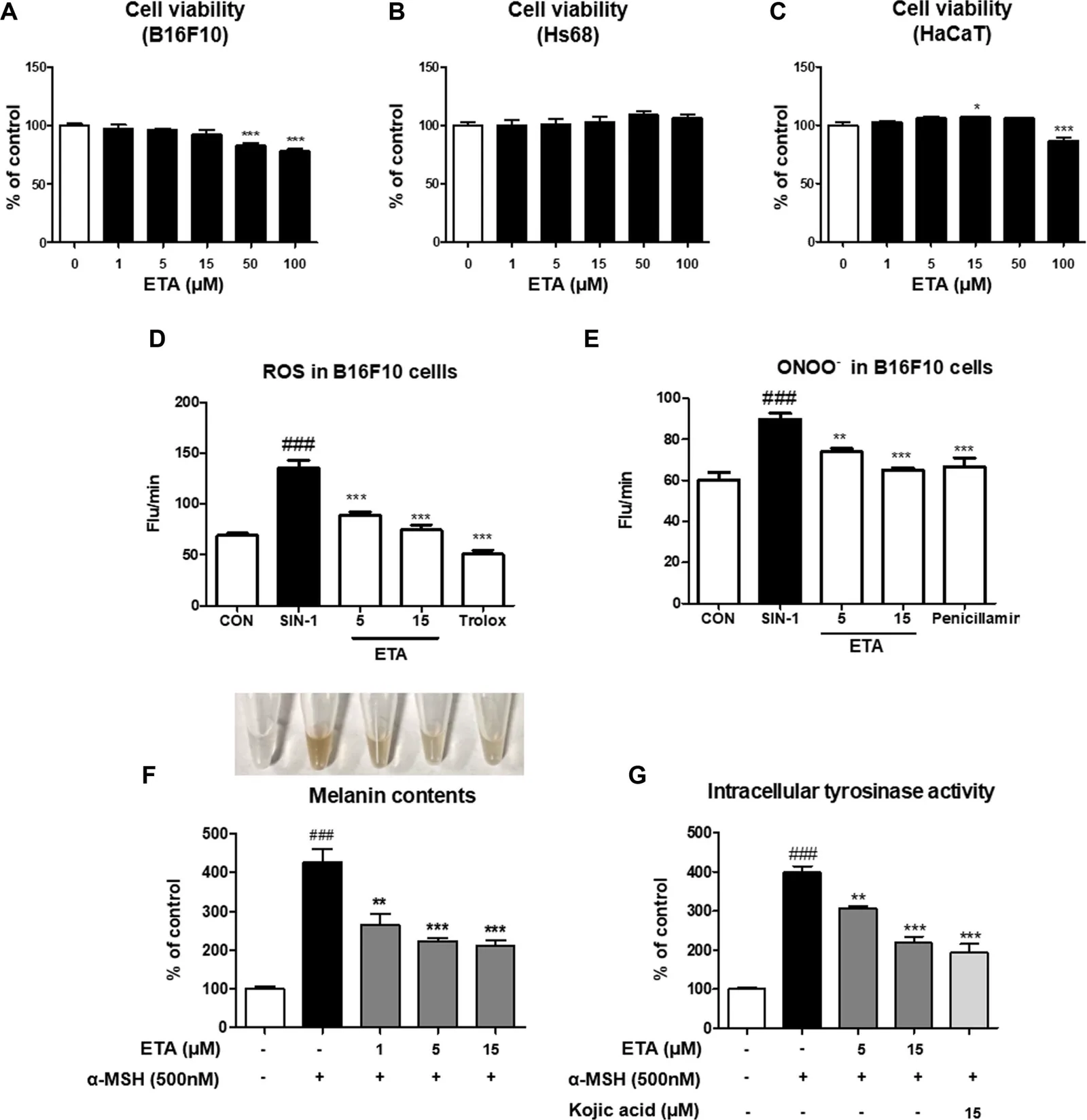
Effects of ETA on melanogenesis in α-MSH-stimulated B16F10 cells. (**A-C**) Cell viability of B16F10, HS68, and HaCaT cells treated with ETA (1–100 μM) for 24 h (n = 6). (**D**) Intracellular reactive oxygen species (ROS) levels in B16F10 cells treated with ETA (5 and 15 μM) for 24 h, followed by SIN-1 (10 μM) treatment for 1 h (n = 10). (**E**) Intracellular peroxynitrite (ONOO^−^) levels in B16F10 cells treated with ETA (5 and 15 μM) for 24 h, followed by SIN-1 (10 μM) treatment for 1 h (n = 10). (**F**) Intracellular melanin content in B16F10 cells pretreated with ETA (1 ,5 and 15 μM) followed by α-MSH (500 nM) stimulation for 6 days (n = 3). (**G**) Intracellular tyrosinase activity in B16F10 cells pretreated with ETA (5 and 15 μM) or kojic acid (15 μM) for 1 h, followed by α-MSH (500 nM) stimulation for 6 days (n = 3). Data are presented as mean ± SEM. Statistical significance was determined by one-way ANOVA followed by Tukey’s multiple comparisons test. # *p* < 0.05 and ### *p* < 0.001 vs. untreated control or SIN-1-treated group, as appropriate; * *p* < 0.05, ** *p* < 0.01, and *** *p* < 0.001 vs. SIN-1- or α-MSH-treated group, as appropriate.

**Fig. 4:**
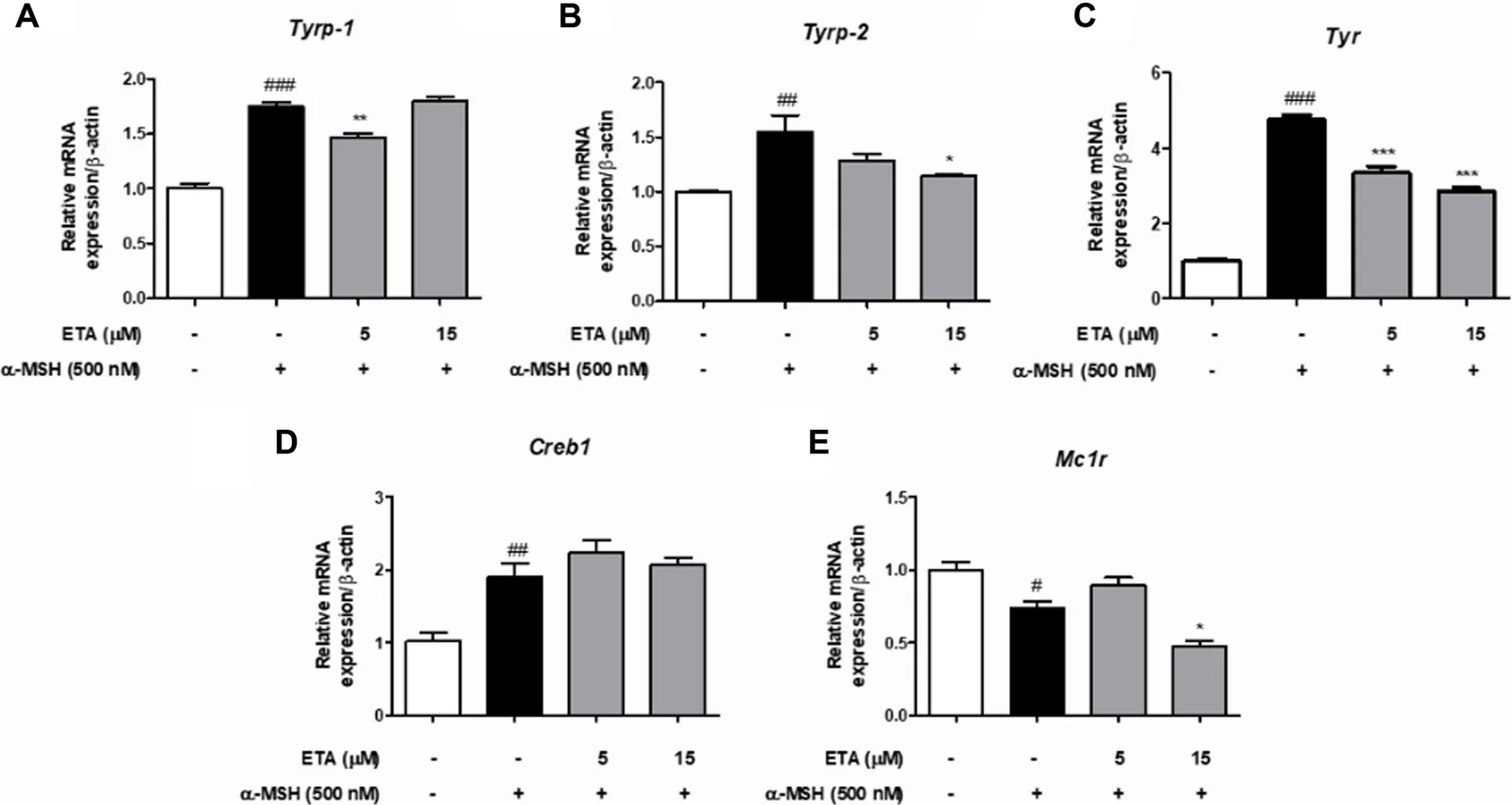
Effects of ETA on melanogenesis-related gene expression in α-MSH-stimulated B16F10 cells. B16F10 cells were treated with ETA at the indicated concentrations and stimulated with α-MSH. The mRNA expression levels of (**A**) *Tyrp1*, (**B**) *Tyrp2*, (**C**) *Tyr*, (**D**) *Creb1*, and (**E**) *Mc1r* were analyzed by quantitative real-time PCR. Data are presented as mean ± SEM (n = 3 independent biological replicates per group). Statistical significance was determined by one-way ANOVA followed by Tukey’s multiple comparison test (# *p* < 0.05, ## *p* < 0.01, ### *p* < 0.001 vs. untreated control; * *p* < 0.05, ** *p* < 0.01, *** *p* < 0.001 vs. α-MSH-treated group).

**Fig. 5:**
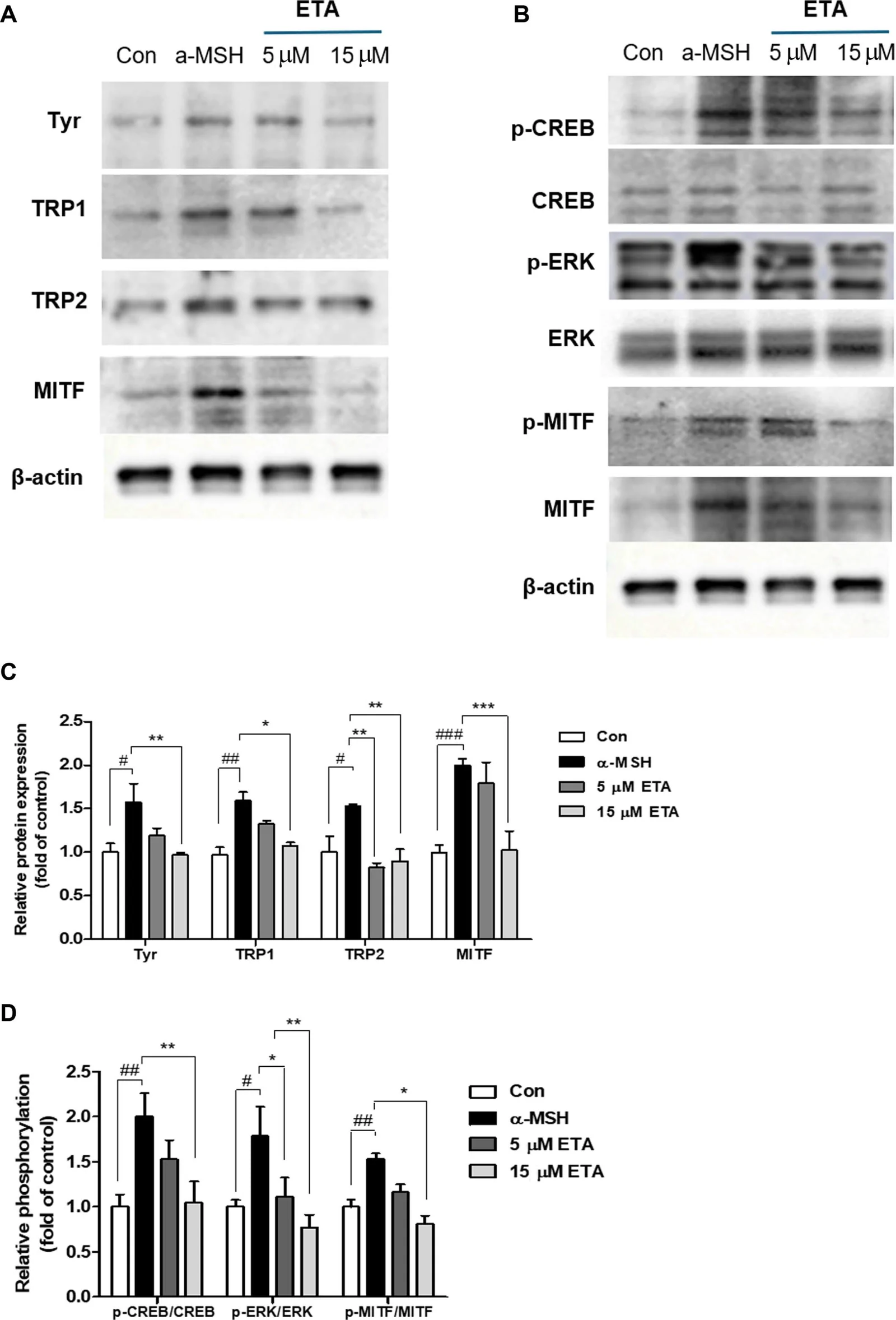
Effects of ETA on melanogenesis-related protein expression and phosphorylation signaling in α-MSH-stimulated B16F10 cells. B16F10 cells were pretreated with ETA at the indicated concentrations for 1 h and then stimulated with α-MSH for 4 days. (**A**) Representative Western blot images of melanogenesis-related proteins, including tyrosinase, TYRP1, TYRP2, and MITF. β-Actin was used as a loading control. (**B**) Representative Western blot images of phosphorylated and total signaling proteins, including p-CREB/CREB, p-ERK/ERK, and p-MITF/MITF. β-Actin was used as a loading control. (**C**) Quantification of tyrosinase, TYRP1, TYRP2, and MITF protein expression normalized to β-actin. (**D**) Quantification of p-CREB, p-ERK, and p-MITF levels normalized to their corresponding total proteins. Band intensities were quantified using ImageJ software (version 1.53e; NIH, USA). Data are presented as mean ± SEM (n = 3 independent experiments). Statistical significance was determined by one-way ANOVA followed by Tukey’s multiple comparison test. # *p* < 0.05, ## *p* < 0.01, and ### *p* < 0.001 vs. untreated control; * *p* < 0.05, ** *p* < 0.01, and ***p < 0.001 vs. α-MSH-treated group.

**Fig. 6:**
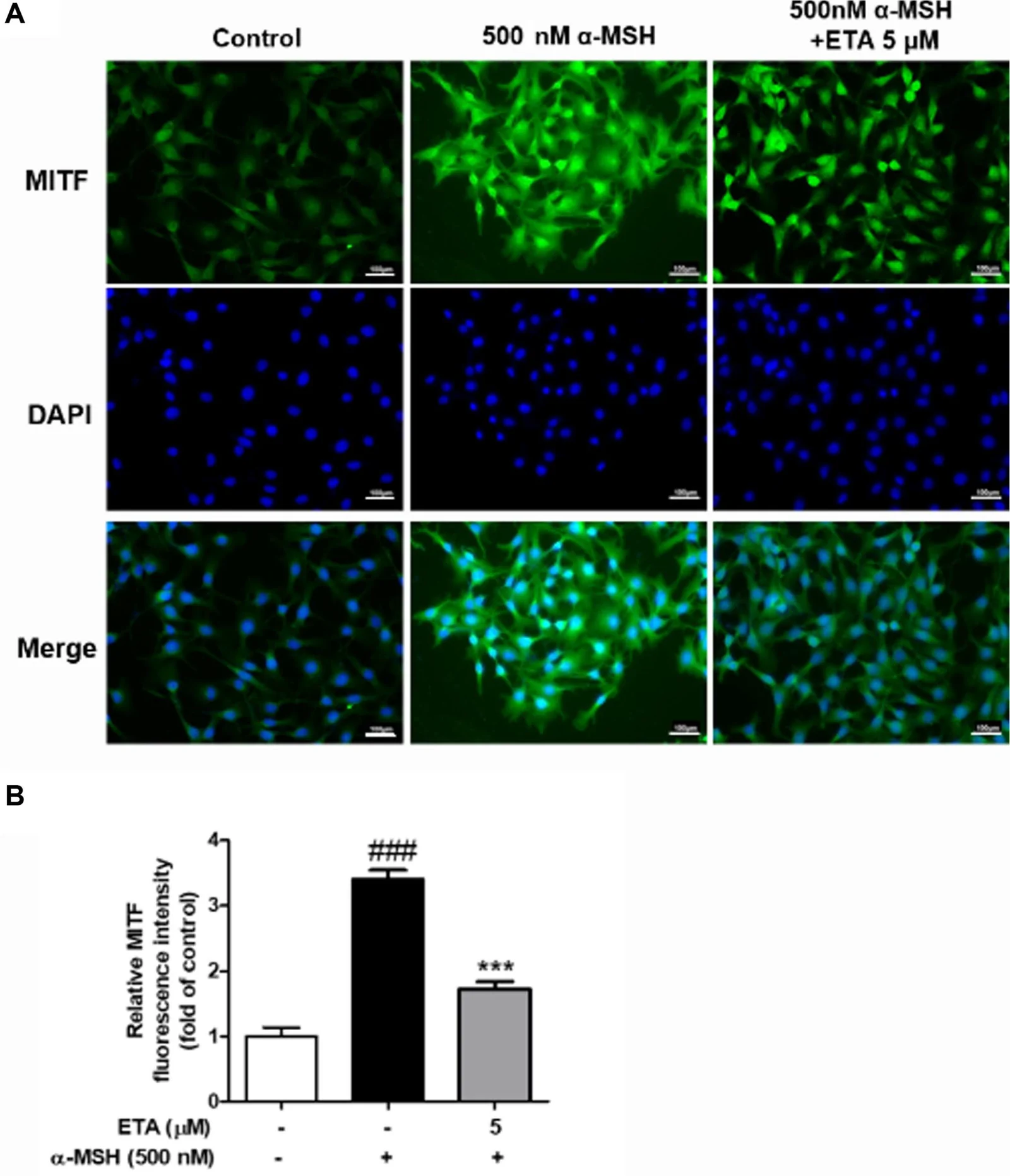
Effects of ETA on MITF fluorescence intensity in α-MSH-stimulated B16F10 cells. B16F10 cells were treated with ETA at the indicated concentration and stimulated with α-MSH. (**A**) Immunofluorescence staining was performed to visualize MITF (green), and nuclei were counterstained with DAPI (blue). Merged images are shown in the lower panels. Scale bar = 100 μm. (**B**) Nuclear MITF fluorescence intensity was quantified using ImageJ software. DAPI-positive nuclear regions were used as regions of interest, and MITF fluorescence intensity within the nuclear area was measured after background subtraction. Data are presented as mean ± SEM from three independent experiments. ### *p* < 0.001 vs. untreated control; *** *p* < 0.001 vs. α-MSH-treated group.

**Table 1 T1:** **Primer sequences used in this study.** Specific primer sets for *Creb1, Mc1r, Tyrp1, Tyrp2,* and *Tyr* are summarized below. To achieve precise target gene amplification, sequences were optimized based on NCBI database information for subsequent molecular applications.

Genes	NCBI Gene ID	Direction	Sequence (5′ → 3′)
Tyrp-1	22178	Forward	GCT GCA GGA GCC TTC TTT CTC
		Reverse	GTC ATC AGT GCA GAC ATC GC
Tyrp-2	104042	Forward	CTC AGA GCTCGG GCT CAG TT
		Reverse	TGT TCA GCA CGC CAT CCA
Tyr	22173	Forward	TTC TGC CTT GGC ACA GAC TT
		Reverse	GCA AGC TGT GGT AGT CGT CT
Mc1r	17199	Forward	CGT CTT GGC TTC CTC TAT G
		Reverse	TGC TAC AGT GTA TGG ATG AAC
Creb1	12912	Forward	GCA ACA TCA TCT GCT CCC AC
		Reverse	TCC CTT CGT TTT TGG GAA TC
