# Supplementary material for: Eicosa-11Z,14Z,17Z-Trienoic Acid Suppresses Melanogenesis by Inhibiting Intracellular MITF/Tyrosinase Axis
Source: J Microbiol Biotechnol. 2026 Oct 8;36:e2604071. doi: 10.4014/jmb.2604.04071 (PMC13686803; doi:10.4014/jmb.2604.04071)
Supplement: Supplementary file 1 [file jmb-36-e2604071-supple.pdf]

J. Microbiol. Biotechnol. 2026. 36: e2604071

https://doi.org/10.4014/jmb.2601.04071

Supplementary Table and Figure

Table S1. Representative unsaturated fatty acid hits retrieved by Swiss Similarity using the Combined screening method.

Compound name	ChEMBL identifier	Fatty acid class	SwissSimilarity score	SMILES
α-Linolenic acid	CHEMBL8739	C18:3 n-3 PUFA	0.999	CC/C=CC/C=CC/C=CCCCCCCCC(=O)O
Linoleic acid	CHEMBL267476	C18:2 n-6 PUFA	0.999	CCCCC/C=CC/C=CCCCCCCCC(=O)O
Dihomo-γ-linolenic acid	CHEMBL465183	C20:3 n-6 PUFA	0.999	CCCCC/C=CC/C=CC/C=CCCCCCCC(=O)O
γ-Linolenic acid	CHEMBL464982	C18:3 n-6 PUFA	0.999	CCCCC/C=CC/C=CC/C=CCCCCC(=O)O
Arachidonic acid	CHEMBL15594	C20:4 n-6 PUFA	0.997	CCCCC/C=CC/C=CC/C=CC/C=CCCCC(=O)O
Eicosapentaenoic acid	CHEMBL460026	C20:5 n-3 PUFA	0.995	CC/C=CC/C=CC/C=CC/C=CC/C=CCCCC(=O)O
Docosahexaenoic Acid	CHEMBL367149	C22:6 n-3 PUFA	0.971	CC/C=CC/C=CC/C=CC/C=CC/C=CC/C=CCCC(=O)O

Representative hits were selected from SwissSimilarity output based on similarity scores and relevance as structurally related unsaturated fatty acids for comparison with ETA. No predefined Tanimoto similarity threshold was applied.

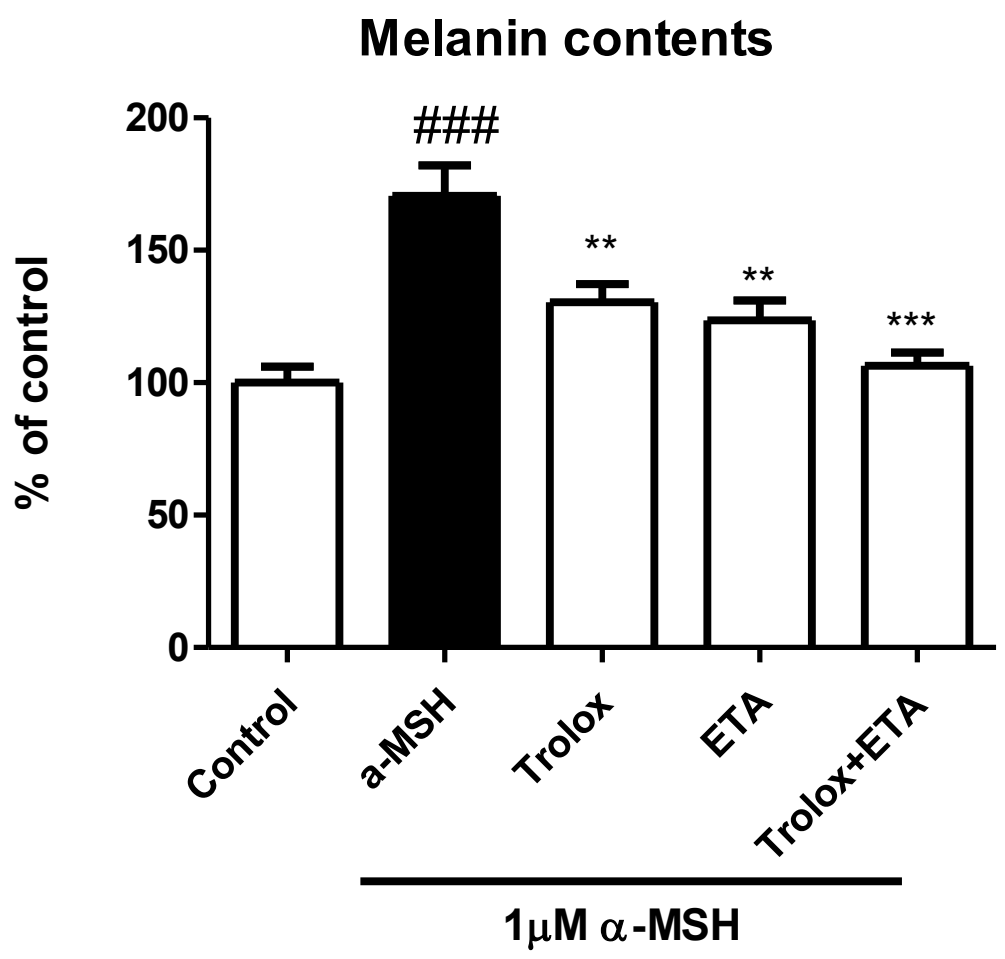

Figure S1. Effect of Trolox co-treatment on ETA-mediated suppression of melanin accumulation in α-MSH-stimulated B16F10 cells.

B16F10 cells were stimulated with α-MSH (1 μM) and treated with Trolox, ETA, or their combination for 5 days. The concentrations of Trolox and ETA were 15 μM and 20 μM, respectively. Melanin content was measured and expressed as a percentage of the untreated control. Co-treatment with Trolox did not reverse the ETA-mediated reduction in α-MSH-induced melanin accumulation. Data are presented as mean ± SEM (n = 4 independent experiments). Statistical significance was determined by one-way ANOVA followed by Tukey's multiple comparison test. ###$p < 0.001$ vs. untreated control; **$p < 0.01$ and ***$p < 0.001$ vs. α-MSH-treated group.
